# Supplementary material for: HIVprotI: an integrated web based platform for prediction and design of HIV proteins inhibitors
Source: J Cheminform. 2018 Mar 9;10:12. doi: 10.1186/s13321-018-0266-y (PMC5845081; doi:10.1186/s13321-018-0266-y)
Supplement: Supplementary file 2 — Additional file 2. Source code of HIVProtI web server. [file 13321_2018_266_MOESM2_ESM.zip › HIVprotI_Source-code/hivprotI/jsmol/ext_rcsb.htm]

RCSB PDB - Ligand Summary


POP-OUT
|
CLOSE

|


  

An Information Portal to Biological Macromolecular Structures

As of
Tuesday Nov 05, 2013 at 4 PM PST
there are
95280 Structures
 | 
PDB Statistics
 |

Print Options:  
  BW  
  Color  |  
  Print

URL:

Everything

Author

Macromolecule

Sequence | options

Ligand | options


Search History,
Previous Results

Search

  
Advanced  
Browse

- |  |  |
  | --- | --- |
  | **PDB-101** | Hide |

  - Structural View of Biology
  - Understanding PDB Data
  - Molecule of the Month
  - Educational Resources
  - Author Profiles
- |  |  |
  | --- | --- |
  | **MyPDB** | Hide |

  - Login to your Account
  - Register a New Account
  - MyPDB Help Page
  - Query Results
  - Query History

  Username:   
     
  Password:   
     

  - Forget your Password?

  **Welcome !**

  - Saved Query Manager
  - Personal Annotations
  - User Account
  - Logout

  **Admin Menu:**

  - Users
  - Groups
  - Status
  - Properties
  - Sessions
  - Loaders
  - Update
  - Load Results
  - Structure Utils
  - Reconcile
  - Missing Images
  - Staged Content
  - Weekly Update
  - Developer Guide
- |  |  |
  | --- | --- |
  | **Home** | Hide |

  - News & Publications
  - Usage/Reference Policies
  - Deposition Policies
  - Website FAQ
  - Deposition FAQ
  - Contact Us
  - About Us
  - Careers
  - External Links
  - Sitemap
  - New Website Features
- |  |  |
  | --- | --- |
  | **Deposition** | Hide |

  - All Deposit Services
  - Electron Microscopy
  - X-ray | NMR
  - Validation Server
  - BioSync Beamlines/Facilities
  - Related Tools
- |  |  |
  | --- | --- |
  | **Tools** | Hide |

  - Download Files
  - Compare Structures
  - Drug & Drug Target Mapping
  - File Formats
  - RESTful Web Services
  - Widgets
- |  |  |
  | --- | --- |
  | **Help** | Hide |

  - Launch Help System
  - Display Settings
  - Video Tutorials
  - Glossary of Terms
  - RCSB PDB *Mobile*

This browser is either not Javascript enabled or has it turned off.  
This site will not function correctly without Javascript.


|  |  |
| --- | --- |
| PROTOPORPHYRIN IX CONTAINING FE   HEM is found in 3177 entries | HEM  Display Files   close  Structure Data File   mmCIF component definition  Download Files   close  Structure Data File  mmCIF component definition  Share this Page  close  Facebook Twitter LinkedIn Email   Link this Page |
| - **Chemical Component Summary**   \| Hide Name \| PROTOPORPHYRIN IX CONTAINING FE; Identifiers \| 3,3'-(7,12-diethenyl-3,8,13,17-tetramethylporphyrin-2,18-diyl)dipropanoic acid 3-[(5Z,10Z,14Z,19Z)-18-(2-carboxyethyl)-8,13-bis(ethenyl)-3,7,12,17-tetramethyl-21,23-dihydroporphyrin-2-yl]propanoic acid; Synonyms \| HEME; Formula \| C_3__4_ H_3__2_ Fe N_4_ O_4_; Molecular Weight \| 616.49 g/mol; Type \| non-polymer; Isomeric SMILES \| CC1=C(CCC(O)=O)C2=Cc3c(CCC(O)=O)c(C)c4C=C5C(C)=C(C=C)C6=[N]5[Fe]5([N]2=C1C=c1c(C=C)c(C)c(=C6)n51)n34; InChI \| InChI=1S/C34H34N4O4/c1-7-21-17(3)25-13-26-19(5)23(9-11-33(39)40)31(37-26)16-32-24(10-12-34(41)42)20(6)28(38-32)15-30-22(8-2)18(4)27(36-30)14-29(21)35-25/h7-8,13-16,36-37H,1-2,9-12H2,3-6H3,(H,39,40)(H,41,42)/b25-13-,26-13-,27-14-,28-15-,29-14-,30-15-,31-16-,32-16-; InChI key \| FEDYMSUPMFCVOD-UJJXFSCMSA-N The chemistry of this component is ambiguous (group, ion, metal cluster etc.) - **Links** \| Hide BindingDB   \| Searches for more than 90% similar small molecules in BindingDB, a public database of measured binding affinities of drug-like molecules with protein drug-targets; ChEBI   \| Searches ChEBI, a freely available dictionary of molecular entities that incorporates an ontological classification; ChemSpider   \| Searches ChemSpider, a free access service providing a structure centric community for chemists; CSLS   \| Searches the Chemical Structure Lookup Service (CSLS), meant to work as an address book for chemical structures; eMolecules   \| Searches eMolecules providing suppliers and information for chemicals; HIC-Up \| A freely accessible resource for structural biologists who are dealing dealing with hetero-compounds ("small molecules"); KEGG COMPOUND   \| Searches KEGG COMPOUND for chemical substances and reactions that are relevant to life; Ligand Expo \| An RCSB PDB resource for searching, exploring, and downloading information and coordinates about the chemical components found in the PDB; PDBeChem \| Dictionary of chemical components in the PDB; PubChem   \| Searches PubChem, a component of NIH's Molecular Libraries Roadmap Initiative with information on the biological activities of small molecules; SuperLigands \| An encyclopedia dedicated to a ligand oriented view that integrates different information about drug-likeness or binding properties; SuperHapten   \| Searches for similar small molecules in SuperHapten, an immunogenic compound database | **Ligand Image** Drag the right-bottom corner to resize. **Toggle** Hydrogens Labels Large image - View atom labels - **Instances in PDB Entries** \| Hide In polymers: 1 entries Ex: 3TNK As free ligands: 3177 entries Ex: 101M, 102M, 103M... Found in 3177 entries total. - **Related Ligands in the PDB** \| Hide Find stereoisomers Find similar ligands Use HEM for a chemical structure search - **Chemical Details** \| Hide Formal Charge \| 0; Atom Count \| 75; Chiral Atom Count \| 1; Chiral Atoms \| FE; Bond Count \| 82; Aromatic Bond Count \| 28 |

The RCSB PDB is managed by two members of the RCSB:   
Rutgers and UCSD, and is funded by NSF,
NIGMS, DOE, NLM,
 NCI,
NINDS, and NIDDK.

© RCSB Protein Data Bank
